# Delayed Time-to-Treatment of an Antisense Morpholino Oligomer Is Effective against Lethal Marburg Virus Infection in Cynomolgus Macaques

**DOI:** 10.1371/journal.pntd.0004456

**Published:** 2016-02-22

**Authors:** Travis K. Warren, Chris A. Whitehouse, Jay Wells, Lisa Welch, Jay S. Charleston, Alison Heald, Donald K. Nichols, Marc E. Mattix, Gustavo Palacios, Jeffrey R. Kugleman, Patrick L. Iversen, Sina Bavari

**Affiliations:** 1 Molecular and Translational Sciences Division, United States Army Medical Research Institute of Infectious Diseases, Frederick, Maryland, United States of America; 2 Sarepta Therapeutics, Inc., Cambridge, Massachusetts, United States of America; 3 Pathology Division, United States Army Medical Research Institute of Infectious Diseases, Frederick, Maryland, United States of America; 4 Center for Genome Sciences, United States Army Medical Research Institute of Infectious Diseases, Frederick, Maryland, United States of America; Centers for Disease Control and Prevention (CDC), UNITED STATES

## Abstract

Marburg virus (MARV) is an Ebola-like virus in the family *Filovirdae* that causes sporadic outbreaks of severe hemorrhagic fever with a case fatality rate as high as 90%. AVI-7288, a positively charged antisense phosphorodiamidate morpholino oligomer (PMO*plus*) targeting the viral nucleoprotein gene, was evaluated as a potential therapeutic intervention for MARV infection following delayed treatment of 1, 24, 48, and 96 h post-infection (PI) in a nonhuman primate lethal challenge model. A total of 30 cynomolgus macaques were divided into 5 groups of 6 and infected with 1,830 plaque forming units of MARV subcutaneously. AVI-7288 was administered by bolus infusion daily for 14 days at 15 mg/kg body weight. Survival was the primary endpoint of the study. While none (0 of 6) of the saline group survived, 83–100% of infected monkeys survived when treatment was initiated 1, 24, 48, or 96 h post-infection (PI). The antisense treatment also reduced serum viremia and inflammatory cytokines in all treatment groups compared to vehicle controls. The antibody immune response to virus was preserved and tissue viral antigen was cleared in AVI-7288 treated animals. These data show that AVI-7288 protects NHPs against an otherwise lethal MARV infection when treatment is initiated up to 96 h PI.

## Author Summary

Marburg virus (MARV) is a filovirus closely related to Ebola virus and similarly causes hemorrhagic fever in humans. MARV is endemic throughout parts of tropical Africa. Severe outbreaks of Marburg virus disease (MVD) have occurred involving hundreds of human cases. No effective MARV antiviral therapies are available. In this study, we used a positive charged phosphorodiamidate morpholino oligomer (PMO*plus*) targeting the mRNA of the MARV nucleoprotein gene as a medical countermeasure to treat disease in a lethal nonhuman primate model of MVD. The intravenous treatment regimen was well tolerated with no treatment related adverse effects. We showed that when using this antisense treatment, serum virus levels decreased and 83–100% of the animals survived, even when the treatment was delayed as much as 96 hours after infection. None of the untreated animals survived the viral challenge in this model. Our results suggest that antisense therapies, such as PMOs, hold great promise for the treatment of severe viral diseases such as MVD.

## Introduction

Marburg virus (MARV) is a member of the family *Filoviridae* that is highly virulent and causes hemorrhagic fever in humans and nonhuman primates [1]. In humans, Marburg virus disease (MVD) typically begins with high fever accompanied by a severe headache, chills, myalgia and malaise. For many patients, this is followed by abdominal pain, severe nausea, vomiting and watery diarrhea. After about 5 days, more than 75% of the patients develop some form of hemorrhagic manifestation such as mucosal bleeding, bloody diarrhea, hematemesis, and ecchymosis [2]. Later stages of the disease can manifest in multiorgan failure, shock, and coma. Acute liver failure, with concomitant increases in liver-associated enzymes, is a common disease manifestation [3]. Case fatality rates can approach 90%, with death typically occurring 8–16 days following the onset of symptoms [1,2].

MARV outbreaks most often occur in sub-Saharan Africa, where they pose a public health threat to the African population and travelers to the region. This threat has been exemplified by the occurrence of two large outbreaks in the Democratic Republic of the Congo in 1998–2000 and in Angola in 2004–2005, which together resulted in greater than 400 cases with more than 350 deaths [1]. MARV is closely related to the Ebola virus and there is the possibility of larger outbreaks similar to the current Ebola virus disease outbreak in West Africa.

The MARV genome is negative-sense, single stranded RNA of approximately 19 kb encoding seven genes including NP (nucleoprotein), VP35 (polymerase cofactor), VP40 (matrix protein), GP (glycoprotein), VP30 (transcription activator), VP24 (secondary matrix protein) and an RNA-dependent RNA polymerase (L protein). These genes and their products represent potential targets for the development of therapeutic agents. There are currently no licensed vaccines or antiviral therapies for MARV infection, although several approaches are actively being pursued for postexposure therapeutic interventions. Daddario-DiCaprio et al. showed the first complete postexposure protection of nonhuman primates against MARV infection using a live-attenuated recombinant vesicular stomatitis virus vaccine vector expressing the MARV GP [4]. However, the construct was given just 20–30 min after viral challenge and safety and efficacy of this therapeutic modality in human subjects remains unproven. Postexposure administration of recombinant nematode anticoagulant protein c2, a strategy designed to mitigate coagulopathy seen in severe MARV infections, failed to provide substantive protection of infected monkeys [5]. Recent studies using liposome formulated small interfering RNAs (siRNAs) targeting the NP gene of MARV in guinea pig and nonhuman primate models are promising [6,7]. In addition, studies have demonstrated the importance of the MARV NP gene in both viral assembly [8] and viral RNA synthesis [9], highlighting the critical role the NP gene plays in the life cycle of the MARV.

Antisense oligomers, such as AVI-7288, are synthetic molecules designed to interfere with the translation of gene products by sterically blocking mRNA resulting in the inhibition of gene expression [10]. AVI-7288 is designed to prevent viral replication by binding to NP mRNA in a sequence-specific manner, thus sterically blocking translation. Phosphorodiamidate morpholino oligomers (PMOs) are uncharged antisense compounds composed of moieties with 6-sided morpholino bases linked by phosphorodiamidate linkages, as opposed to a ribose base as in RNA, linked through methylene phosphorodiamidate. PMOs are attractive as antiviral agents due to their metabolic stability, and bioavailability [11,12]. PMOs and peptide-conjugated PMOs (PPMO) have been shown to inhibit the replication of Ebola virus [13,14], coxsackievirus B3 [15], influenza A virus [16,17], alphaviruses [18], vesiviruses [19], dengue virus [20], and the severe acute respiratory syndrome-associated coronavirus [21]. In a previous report, we introduced a new class of positively-charged PMO (PMO*plus*) containing piperazine linkages within the molecular backbone and containing a limited number (between 2 and 5) of positive charges [14]. The positive charges may enhance both cellular penetration and avidity to the negatively charged RNA target sequence of the virus. We previously showed that administration of PMO*plus*, in a combination therapy containing antisense agents targeting NP and VP24 of MARV, provided 100% protection of cynomolgus macaques when given 30–60 min after infection [22]. Subsequently, we showed that a single agent designated AVI-7288, targeting the NP of MARV, was as effective as the combination therapy [23]. In the present study, we evaluated the efficacy of AVI-7288 alone in cynomolgus macaques in a delayed time-to-treatment study in which the PMO*plus* was administered 1, 24, 48, or 96 h following infection with MARV.

## Materials and Methods

### Ethics Statement

Animal research at U.S. Army Medical Research Institute of Infectious Diseases (USAMRIID) was conducted under an Institutional Animal Care and Use Committee (IACUC) approved protocol in compliance with the Animal Welfare Act, PHS Policy, and other federal statutes and regulations relating to animals and experiments involving animals. The facility where this research was conducted is accredited by the Association for Assessment and Accreditation of Laboratory Animal Care, International and adheres to principles stated in the *Guide for the Care and Use of Laboratory Animals*, National Research Council, 2011[24].

### Animals and Animal Experiments

Thirty (23 male and 7 female) healthy cynomolgus macaques (*Macaca fascicularis*) were obtained from Primate Products, Inc. (Immokalee, FL). Animals were housed individually in stainless steel cages with enrichment toys, and they were provided fruit, monkey chow biscuits and water *ad libitum*. Animals were sedated for blood collection procedures using ketamine (100 mg/mL)/acepromazine (10 mg/mL) at 0.1 mL/kg administered intramuscularly. Four days before viral challenge, a blood sample was collected for determination of “baseline” values for each animal’s serum chemistry values, hematology, and coagulation parameters. The macaques were randomized into five groups with six animals (including at least one female) in each group. Animals were challenged with 1,830 plaque forming units (PFU) of MARV Musoke by subcutaneous injection. AVI-7288 was administered once daily for 14 d by bolus IV injection at a dose of 15 mg/kg beginning approximately 1, 24, 48, or 96 h after virus challenge. An infection-control group received IV saline (0.9% NaCl) according to the same regimen as AVI-7288, beginning 1 h after challenge. Table 1 summarizes the animal groupings and treatment regimens. All MARV-infected animals were handled under maximum containment in a biosafety level 4 (BSL-4) laboratory at USAMRIID in Frederick, Maryland.

**Table 1:** Study design.

Group	Treatment	PMO*plus* dose (mg/kg/day)	Regimen	# of Males/Females	Time-to-treatment post infection (h)
1	AVI-7288	15	SID; D0-D13	4/2	1
2	AVI-7288	15	SID; D1-D14	5/1	24
3	AVI-7288	15	SID; D2-D15	5/1	48
4	AVI-7288	15	SID; D4-D17	5/1	96
5	PBS	0	SID; D0-D13	4/2	1

SID = once per day

D0 = day of viral challenge; D1 = 1 day after viral challenge, etc.

After viral challenge, all macaques were monitored closely for signs of clinical disease and euthanasia was performed according to pre-specified criteria [25]. Blood samples were collected from each animal on day 3, 5, 8, 10, 14, 21, 28, and 41 PI. Macaques that survived until day 41 were considered to have survived the viral challenge.

### Cells and Viruses

Vero cells were obtained from the American Type Culture Collection (ATCC) and maintained in Dulbecco's Modified Eagle Medium (Life Technologies, USA) supplemented with 10% fetal bovine serum (FBS, Life Technologies, USA). The Marburg virus (Marburg virus H. sapiens-tc/KEN/1980/Musoke) used in these studies was originally isolated from the serum of an infected patient [26] and was propagated through a total of six passages in Vero cells.

### PMO*plus*

The positively charged PMO (PMO*plus*) contains piperazine linkages within the molecular backbone and was designed with sequence homology overlapping the AUG translational start site of MARV NP gene. This PMO*plus* was designated AVI-7288 and its sequence is 5’-GAATATTAAC+AI+AC+TGAC+A+AGTC-3’, where the “+” represents the positively charged piperazine moieties [27]. AVI-7288 was synthesized by Sarepta Therapeutics (Cambridge, MA) as previously described [27].

### Viral Plaque Assays

Serum samples were 10-fold serially diluted in Eagle’s Essential Minimal medium and were added to duplicate wells of Vero cell monolayers in 6-well plates. After 7–9 d incubation, a secondary agarose overlay containing neutral red was applied to each well for 24–48 h to aid in plaque visualization. The lower limit of quantification for this assay was 1,000 PFU/mL.

Tissues collected for viral load assessment were collected and stored at -60 to -80°C until analysis. These included adrenal glands, femoral bone marrow, brain, heart, kidney, liver, lungs, lymph nodes, spleen, and testes/ovaries. Prior to use in the assay, tissues were homogenized in cell culture medium at a ratio of 0.1g of tissue to 1.0 mL of medium using a hand-held homogenizer with disposable rotor, and the homogenate was subjected to plaque assay as described for serum.

### Quantitative RT-PCR

Total RNA was extracted from TRIzol LS-treated serum samples using MagMax 96 Blood RNA Isolation Kit (Ambion) and purified RNA samples were stored at -20°C until assayed. The qRT-PCR assays were performed on samples in triplicate using an ABI PRISM 7900HT Sequence Detection System with RNA UltraSenseTM one-step Kit (Invitrogen) and TaqMan Probe (ABI) in accordance with the manufacturer’s instructions. MARV Musoke-specific primers MARV_GP2_F (5’-TCACTGAAGGGAACATAGCAGCTAT-3’), MARV_GP2_R (5’-TTGCCGCGAGAAAATCATTT-3’), and probe MARV_GP2_P (6FAM–ATTGTCAATAAGACAGTGCAC-MGB) were used in each reaction. At least six serial 10-fold dilutions of quantified MARV genomic RNA were used to generate a standard curve. Quantification of genome copies per volume serum were calculated from the cycle-threshold value obtained for each sample compared using the standard-curve equation. The lower limit of quantification for this analysis was 1.3 x 10^5^ genomic equivalents/mL of serum.

### Viral Genome Sequence Analysis

Total RNA from TRIzol LS-treated plasma was used for viral genome sequencing. The quantity of viral RNA present in plasma samples was assessed by qRT-PCR and samples submitted for sequence analysis were ≥ 1.3 x 10^5^ copies/mL. Because of the limitations of viral RNA available for sequence analysis in particular samples, a priority was placed on determining the sequence of the viral genome at bases that code for transcript regions targeted by AVI-7288. These priority regions were: nucleotides 73–95 and 10204–10224 of the MARV genome (GenBank Accession #: NC_001608) and approximately 100 nucleotides 5’ and 3’ to these regions.

### Clinical Pathology

Blood chemistry profiles were determined using a Vitros 350 Chemistry system (Ortho Clinical Diagnostics) and coagulation analysis was performed using a Sysmex CA-560 (Siemens).

### Immunologic Analysis

Serum samples were analyzed to determine circulating cytokine/chemokine concentrations using a Meso Scale Discovery SI6000 platform [Meso Scale Diagnostics (MSD), Rockville, MD] according to manufacturer’s instructions. The Human ProInflammatory 10-plex kit (for IL-2, IL-8, IL-12p70, IL-1β, GM-CSF, IFN-γ, IL-6, IL-10, and TNF-α), the Human IFN-α2a Ultra-Sensitive kit, and the Human Chemokine 10-plex kit (for Eotaxin, MIP-1β, Eotaxin-3, TARC, IP-10, IL-8, MCP-1, MDC, MCP-4) were purchased from MSD. All reagents were provided with the MSD kits. Assays were performed according to manufacturer’s recommended procedures.

To assess development of virus-specific humoral immune response analysis, serum samples were subjected to limiting dilution ELISA to evaluate the titer of anti-MARV GP immunoglobulin G (IgG) and IgM. Recombinant MARV GP (Lot# 12941) was obtained from the National Cancer Institute (Frederick, MD). Serum samples were added to the assay in duplicate using a ½-log dilution series beginning with a 1:100 dilution of virus and ending with a 1:320,000 dilution. Anti-human horseradish peroxidase conjugated antibody was used for detection and Sure Blue TM TMB Solution (KPL, Inc.) was used as substrate.

### Histopathology and Immunohistochemistry

Animals were euthanized by intravenously-administered pentobarbital. A full necropsy was conducted on the carcass of each monkey and a sample of the following organs was collected from each animal for determination of tissue viral titer by plaque assay: axillary lymph node, inguinal lymph node, mesenteric lymph node, liver, spleen, adrenal gland, kidney, gonad, heart, lung, femoral bone marrow, and brain. A complete set of tissue samples was also collected from each monkey for analysis by routine histology and immunohistochemistry (IHC).

Tissues collected for histopathology and IHC were immersion-fixed in 10% neutral buffered formalin for a minimum of 21 days before removal from BSL-4 containment. The tissue samples were trimmed, routinely processed, and embedded in paraffin. Sections of the paraffin-embedded tissues 5 μm thick were cut for histology. The histology slides were deparaffinized and stained with hematoxylin and eosin.

Replicate sets of the slides produced for routine histology were made for IHC. An immunoperoxidase assay using a cocktail of two mouse monoclonal antibodies against MARV as the primary antibodies was completed on unstained slides of all tissue sections. These slides were then counter-stained with hematoxylin.

### Statistical Analysis

Animals were randomly assigned to treatment groups stratified by sex and balanced by body weight. Due to safety considerations, study personnel were experimentally blinded, using single-blind conditions, only to the saline-control and 1 h PI treatment groups. Study data were analyzed with a computerized statistical program (SAS Version 9.3). Mean times to death were evaluated using Student’s t-test, and Kaplan-Meier survival analysis was used to calculate survival curves as well as median and mean survival times. Resulting survival curves were compared by Log-rank (Mantel-Cox) test using Prism GraphPad Software (GraphPad Inc, LaJolla, CA.). When sample sizes permitted, physiologic responses were assessed using Student’s t-test or ANOVA.

## Results

### Postexposure Protection of MARV-Infected Macaques by Treatment with AVI-7288

**Fig 1:**
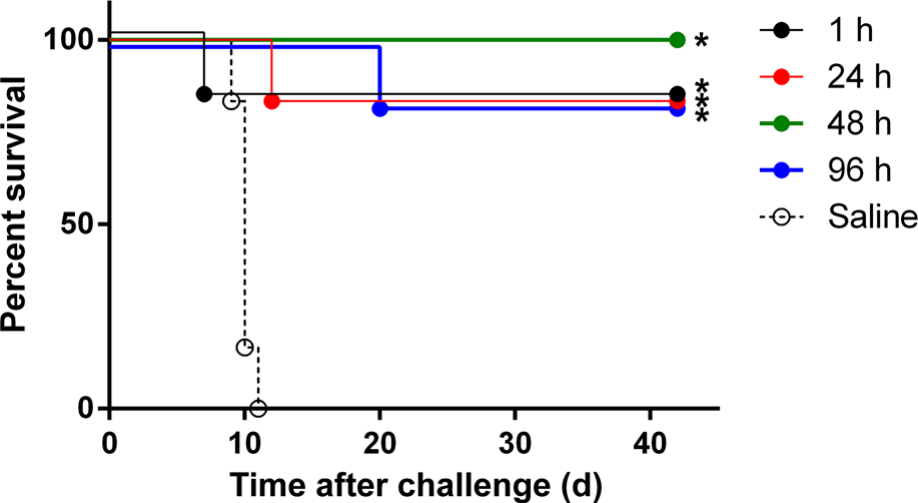
NHP survival after infection. Kaplan-Meier curves of cynomogus macaques (n = 30) infected with MARV-Musoke and then treated once daily for 14 d with bolus IV infection of 15 mg/kg AVI-7288 at 1 (n = 6), 24 (n = 6), 48 (n = 6), or 96 h (n = 6) after virus challenge. Saline-treated animals were used as controls (n = 6). Statistically significant differences (*p<0.0001) between survival curves (Mantel-Cox Log-rank test) of AVI-7288 treatments are indicated.

A total of 30 cynomolgus macaques were challenged with a lethal dose of MARV. Initiation of AVI-7288 treatment occurred at 1, 24, 48, and 96 h after viral infection. Infection controls were administrated saline. One saline-treated monkey died on day 9 and all the animals in this control group succumbed by day 11 PI (Fig 1) after developing characteristic MVD signs such as fever and petechial rash. In contrast, 83% (five of six) of the monkeys in the 1, 24, and 96 h PI treatment groups survived. All of the monkeys (six of six) in the 48 h PI treatment group survived (Fig 1). Although the death of the one animal that died in the 1 h PI treatment group was attributed to complications associated with anesthesia administration, and there were only mild signs of MVD in this monkey, this mortality was treated as a treatment failure and was included in the survival statistics. The differences in survival between the PBS- and AVI-7288-treated groups were highly statistically significant (P < 0.0001), while the differences in survival between groups receiving AVI-7288 treatments were statistically indistinguishable. No significant differences were observed in animal body temperature or weight during the course of the study (S1 Fig). Animals first began to exhibit clinical signs of disease, as measured by their responsiveness and appearance, on day 8 post infection (S2 Fig).

### Viral Load Determinations

**Fig 2:**
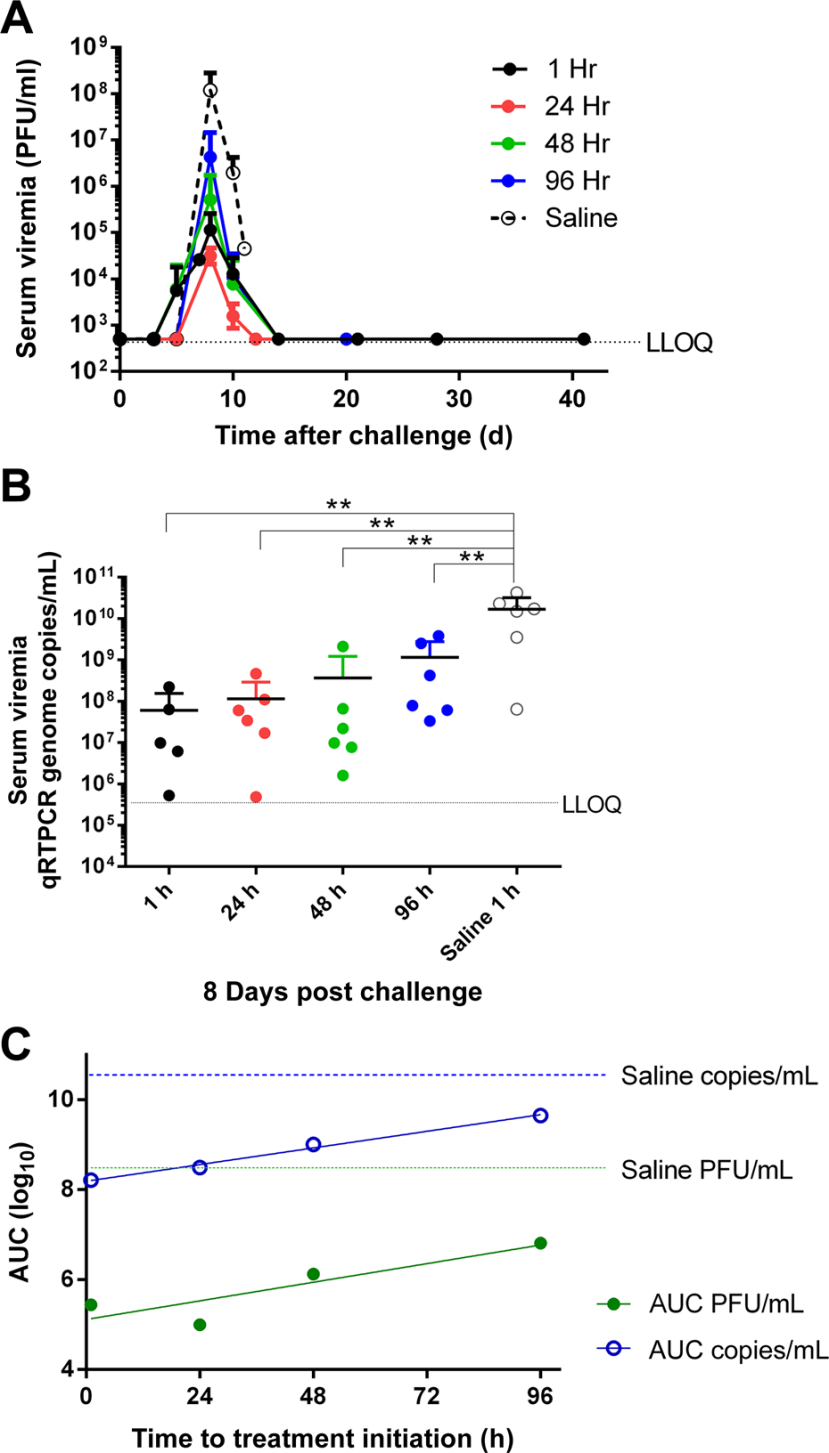
Viral titers and genome equivalents in serum. Mean serum (A) infectious virus and (B) viral RNA concentration in each treatment group. Infectious virus was determined by plaque assay with a lower limit of quantitation of 1,000 PFU/mL, and viral RNA was determined by quantitative RT-PCR with a lower limit of quantitation of 1.33 x 10^5^ genome equivalents/mL. Serum viral RNA on day 8 with the peak serum infectious virus for each animal and the mean and standard deviation indicated by horizontal line and error bar. ANOVA P *<* 0.01 and Tukey’s multiple comparisons significant differences are indicated by (**) for P *=* 0.001. (C) Area under the serum viremia versus time curve on the ordinate versus time to treatment initiation on the abscissa. The blue open circles represent viral RNA/mL and the filled green circles represent PFU. The slope of the genome equivalents/mL is 0.0154 ± 0.00104 and r^2^ of 0.991, P = 0.045. The slope of the PFU/mL is 0.0172 ± 0.006, r^2^ of 0.782, P = 0.116. Values <LLOQ were converted to LLOQ for display and analysis.

Serum viral RNA was first detected at Day 5 in all saline control animals and in approximately 50% of the animals in the treatment cohorts. In all animals, viremia peaked on day 8 PI (Fig 2A and S3 Fig), and these data were consistent with viral genome copy numbers determined by qRT-PCR (Fig 2B and S4 Fig). At the peak of viremia (day 8 PI), the mean viral load in AVI-7288-treated monkeys was reduced approximately 100 times that of saline-treated animals (Fig 2C). For survivors, viral load, measured by plaque assay and qRT-PCR in serum, began to decrease by day 10 PI, had decreased to undetectable levels by day 14, and remained undetectable through the end of the study (41 days).

In saline-treated macaques, virus was detected in nearly all tissues that were analyzed (S5 Fig). The highest viral titer was generally present in the liver, gonads, and spleen (range: 3.6 x 10^6^–4.7 x 10^7^ PFU/g tissue). There was substantial variability in the tissue-specific viral load between individual animals in this group (S5 Fig). While virus was detected in multiple tissues in two of the three AVI-7288-treated animals that succumbed, no virus was detected in any tissues obtained from AVI-7288-treated animals that survived, with the exception of one animal (#4626, 24 h PI treatment group). This animal tested positive for virus in 5 of 12 tissues, including bone marrow, brain, kidneys, inguinal and mesenteric lymph nodes; however, viral titers in these tissues were generally low (range: 1.5 x 10^2^–5.5 x 10^3^ PFU/g tissue) and viral antigen was not detected by immunohistochemistry. The low titers of infectious virus in these tissues and undetectable levels in the remaining survivor tissues indicate the resolution of virus infection.

### Viral Genomic Sequence Analysis

Population genomic analysis of blood-borne MARV revealed that virus from one animal (#9301) in the 24 h PI treatment group acquired a mutation in a region flanking the AVI-7288 binding site. This mutation did not appear to result in a drug-resistant phenotype as the nucleotide change was tolerated and the animal survived infection with MARV. There was no evidence of this mutation seen in virus from any other animals in the study.

### Clinical Pathology

**Fig 3:**
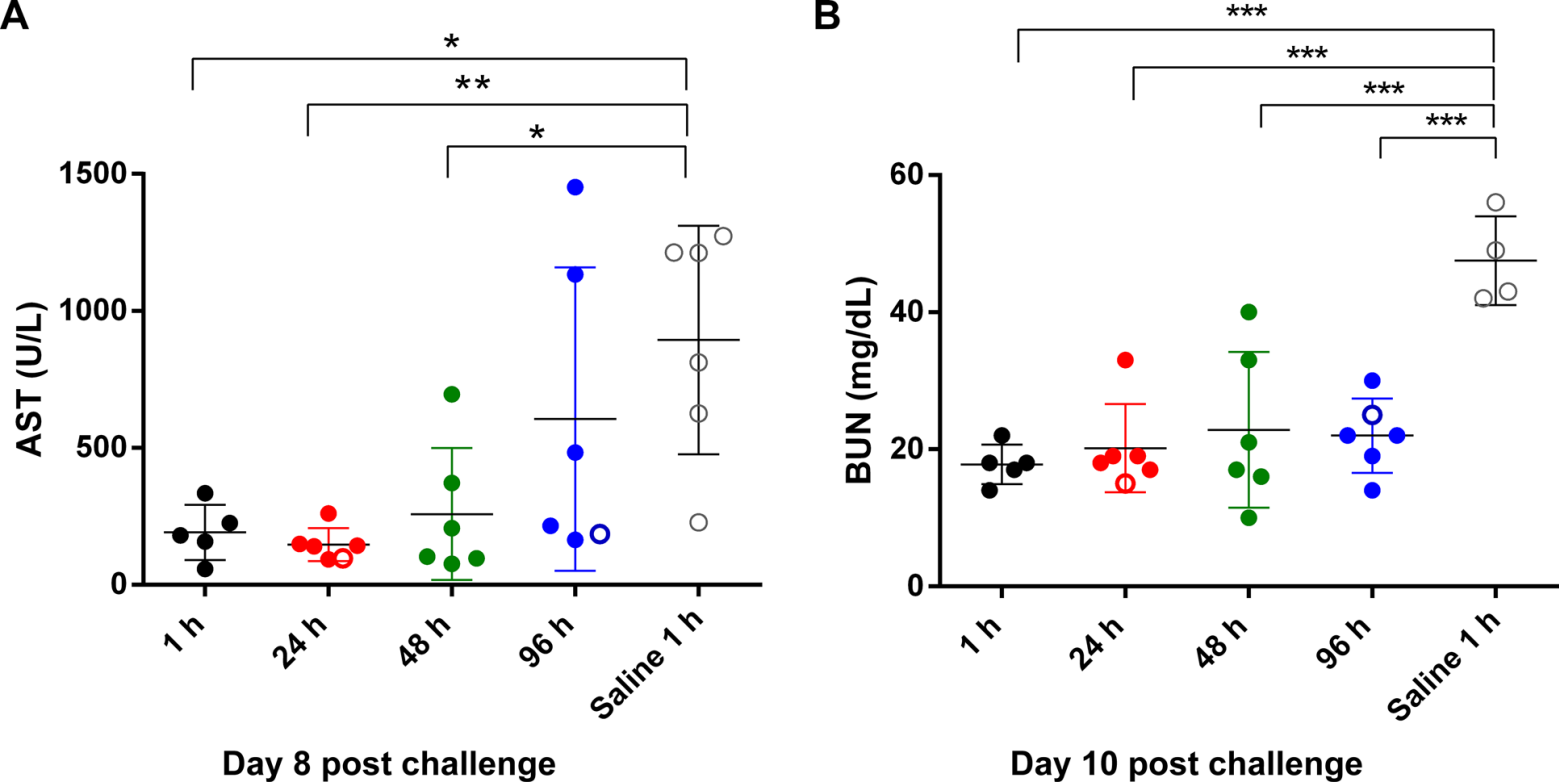
Serum chemistry results. Analysis of the (A) aspartate aminotransferase (AST) and (B) blood urea nitrogen (BUN) for animals on day 10 post challenge. Treatment with AVI-7288 prevented elevation of BUN (P < 0.0001), but treatment did not significantly prevent elevation in ALT (P ≤ 0.206). Open symbols represent animals that succumbed and closed symbols are for survivors. Means are indicated as a horizontal line. Statistically significant differences (*P < 0.05; **P < 0.01; ***P < 0.005) between means of AVI-7288 treatments and control group are indicated.

Liver damage, and subsequent increases in liver enzymes, is a common disease manifestation of VHFs, including that caused by MARV. Many of the animals exhibited marked increases in serum aspartate aminotransferase (AST) by day 5–8 and these elevations resolved by day 28 in all survivors. By day 8, AST was significantly increased in the PBS-treated controls as compared to animals in the 1, 48, and 96 h (P < 0.05) and 24h (P < 0.01) PI groups (Fig 3A). Similar to AST, serum alanine aminotransferase (ALT) levels were increased in all surviving animals by day 8 and resolved to within normal limits by day 28 in most of the survivors (S6 Fig). There were also increases in serum bilirubin levels in animals from all of the groups (S7 Fig). In addition, increases in serum levels of alkaline phosphatase (ALP) and gamma glutamyl transferase (GGT) occurred as early as day 8 in animals from all groups, but resolved to within normal limits by day 21 in most of the survivors; however, some animals from the 96 h PI treatment group had increased ALP and GGT levels that persisted to the end of the study (day 41 PI) (S8 Fig). Increased AST and ALT can occur when hepatocytes are damaged and the accompanying hepatocellular swelling causes cholestasis, which is characterized by hyperbilrubinemia and increases in serum ALP and GGT; taken together, these data are suggestive of hepatocellular damage in the infected animals, with partial to complete resolution in the AVI-7288-treated survivors.

**Fig 4:**
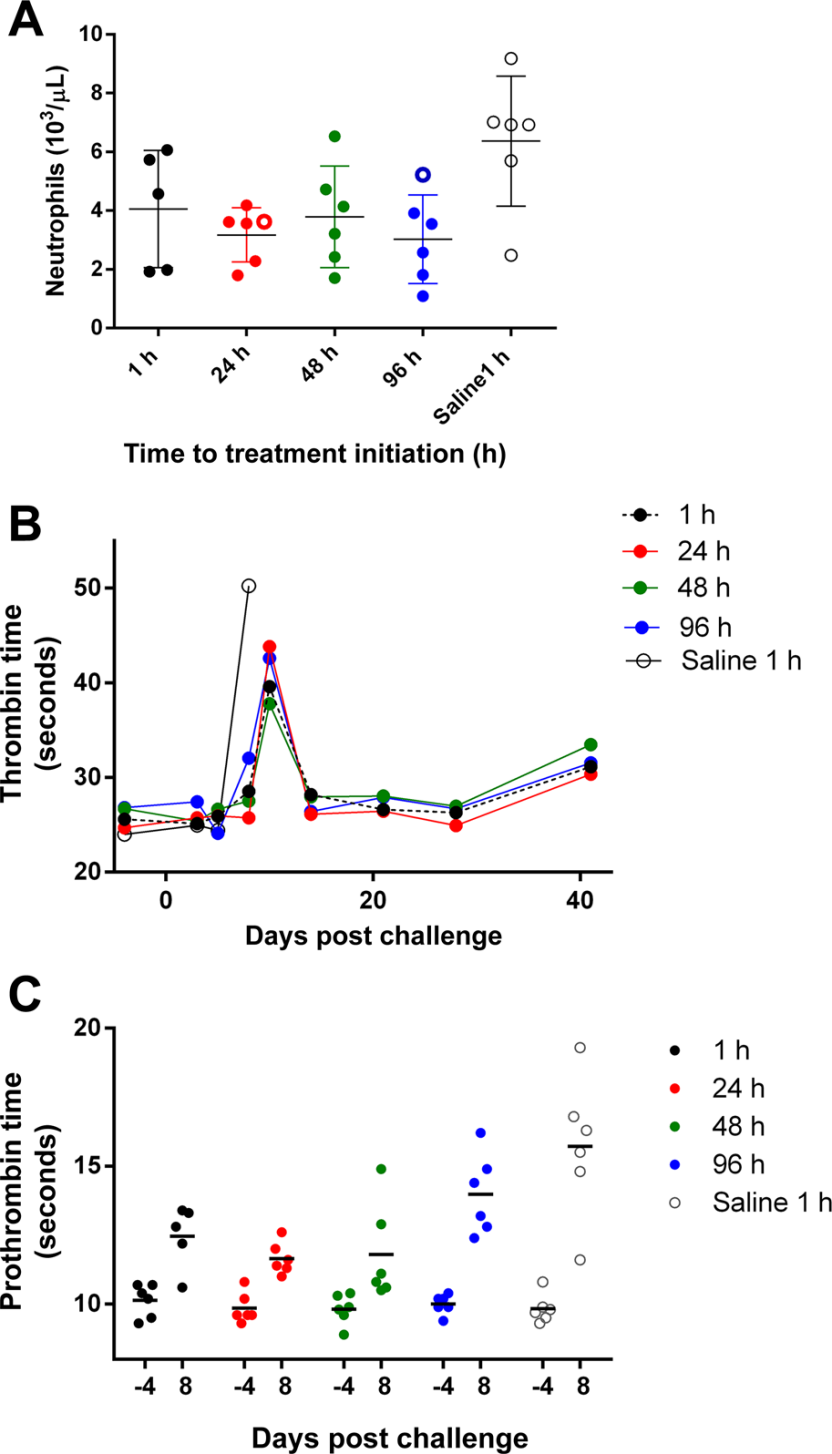
Neutrophil and coagulation results. Analysis of (A) neutrophils on day 8 PI, (B) thrombin time, and (C) prothrombin time. Open symbols represent animals that succumbed and closed symbols are for survivors. Statistically significant differences (*P < 0.05) between means of AVI-7288 treatment groups and saline control group are indicated. Means are indicated as a horizontal line.

On day 10, the blood urea nitrogen (BUN) was significantly increased (P < 0.01) in the four surviving saline-treated controls as compared to animals in all AVI-7288-treatment groups (Fig 3B). Increases in BUN can be indicative of decreased kidney function, which may have been caused by decreased blood flow to the kidneys due to dehydration and/or shock. There was a statistically significant (P < 0.05) increase in neutrophils in macaques in the saline treatment group as compared to the 24 h and 96 h treatment groups, suggesting that viral-induced neutrophilia was prevented in the AVI-7288 treated animals (Fig 4A).

Viral-induced coagulopathy is a common feature of MVD in both humans and nonhuman primates. Consistent with this, decreased platelet counts were observed in animals in all groups (S9 Fig). This decrease was associated with increased MPV, suggesting an adaptive bone marrow response in AVI-7288 treated survivors (S9 Fig). MARV-induced coagulopathy was further evidenced by the prolongation of multiple coagulation parameters, including TT (Fig 4B), PT (Fig 4C and S10 Fig), and activated partial thromboplastin time (APTT; S10 Fig). Surviving animals in the 1, 24, and 48 h PI treatment groups had amelioration of their PT and APTT abnormalities by days 21–28 (S10 Fig).

### Immune Response

**Fig 5:**
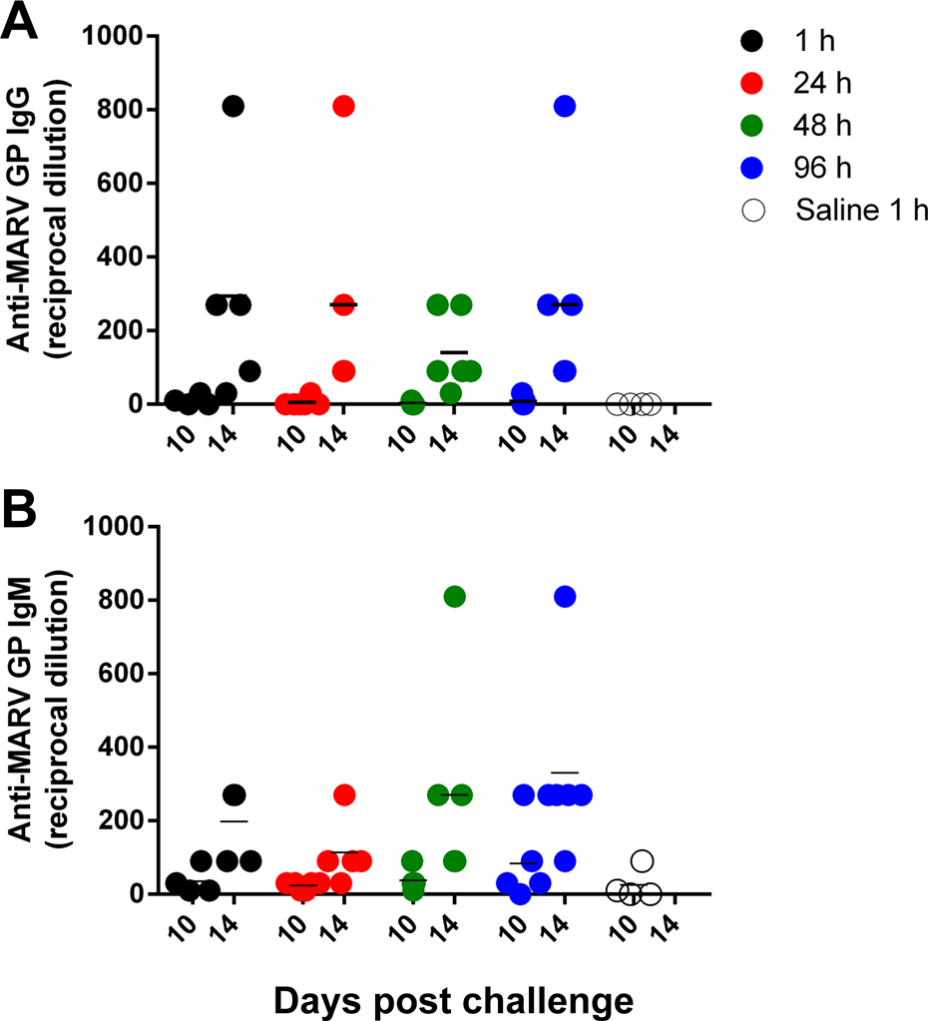
Antibody responses results. MARV-specific anti-GP (A) IgM and (B) IgG antibody titers for each animal at day 10 and day 14 post challenge.

**Fig 6:**
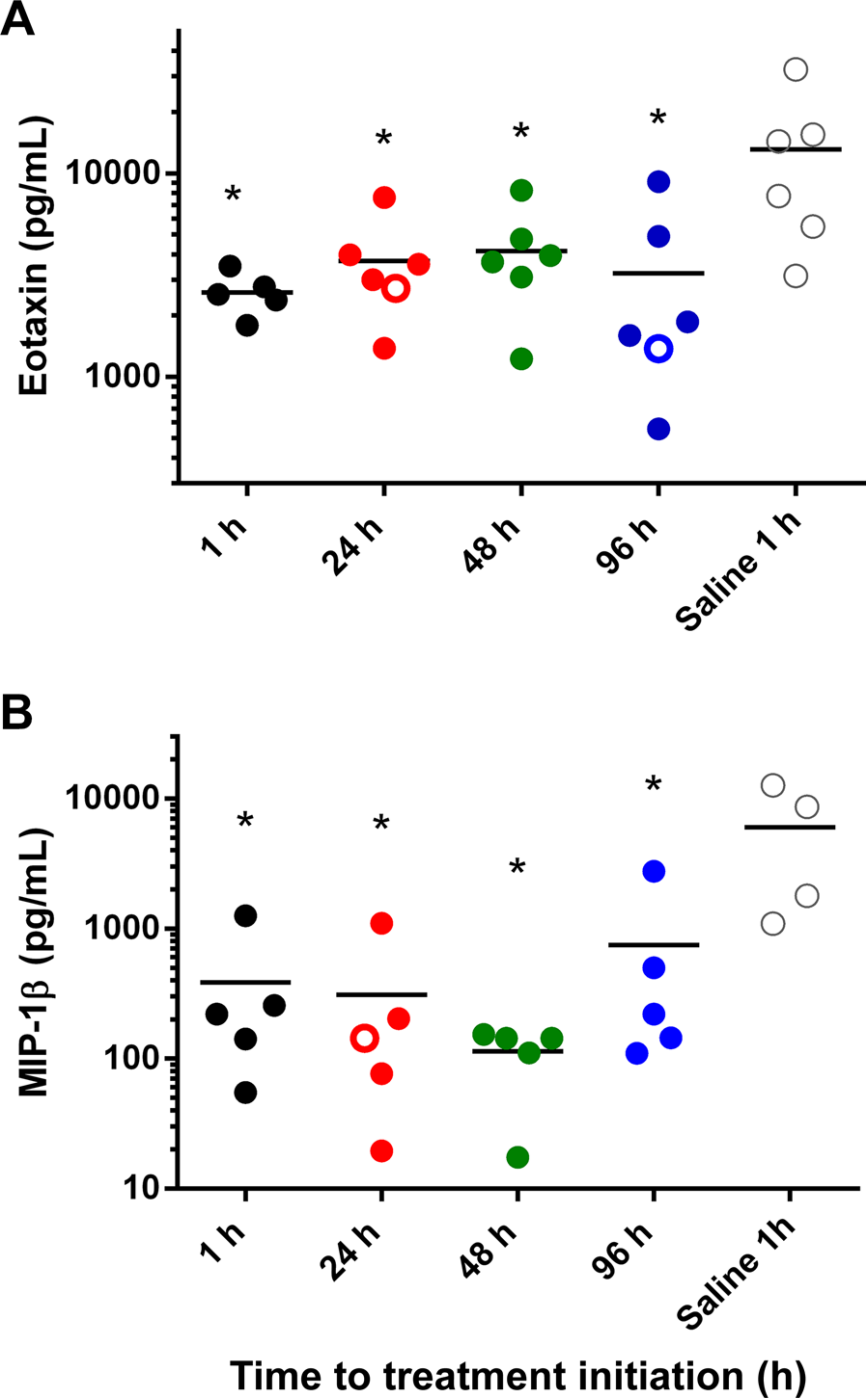
Immune responses results. (A) Eotaxin concentrations on day 8 post infection for each animal. ANOVA indicates significant differences, P = 0.012 and Dunnett’s multiple comparisons test indicates individual treatment groups are each significantly different from saline controls, P < 0.05 indicated with single asterisk. (B) MIP-1β on day 8 post-infection for each animal. ANOVA indicates individual treatment groups are significantly different from saline controls, P = 0.0057 and Dunnett’s multiple comparisons test indicates individual treatment groups are each significantly different from saline controls, P < 0.01 indicated with an asterisk.

MARV-specific IgM was not detected in any of the AVI-7288-treated animals before day 8 PI, at which point it was found in low titers (1:10) in 3 of 29 animals. By day 14 PI, MARV-specific IgM was detected in all surviving animals and generally reached a maximum with titers ranging from 1:30 to 1:810 (Fig 5A). A gradual reduction in IgM was observed in all animals through day 41 PI. MARV-specific IgG was first detected on day 10 PI in 6 of 24 animals (Fig 5B). By day 14 PI, MARV-specific IgG was detected in all surviving animals and titers generally increased from day 14 to day 21 PI and plateaued thereafter through the end of the study with titers ranging from 1:90 to 1:2430. Elevated levels of several proinflammatory chemokines, including Eotaxin, IP-10, and MCP-1, were observed late in infection (peaking at days 8–14 PI) in animals in all treatment groups. However, peak levels of Eotaxin, MCP-1 (Fig 6A), and IP-10 were lower in AVI-7288-treated animals. Saline-treated animals also showed an increased in MDC, TARC, IL-6, and MIP1β prior to death, which on average were higher than observed in the AVI-7288 treated animals (S11 Fig).

### Histopathology and IHC

**Fig 7:**
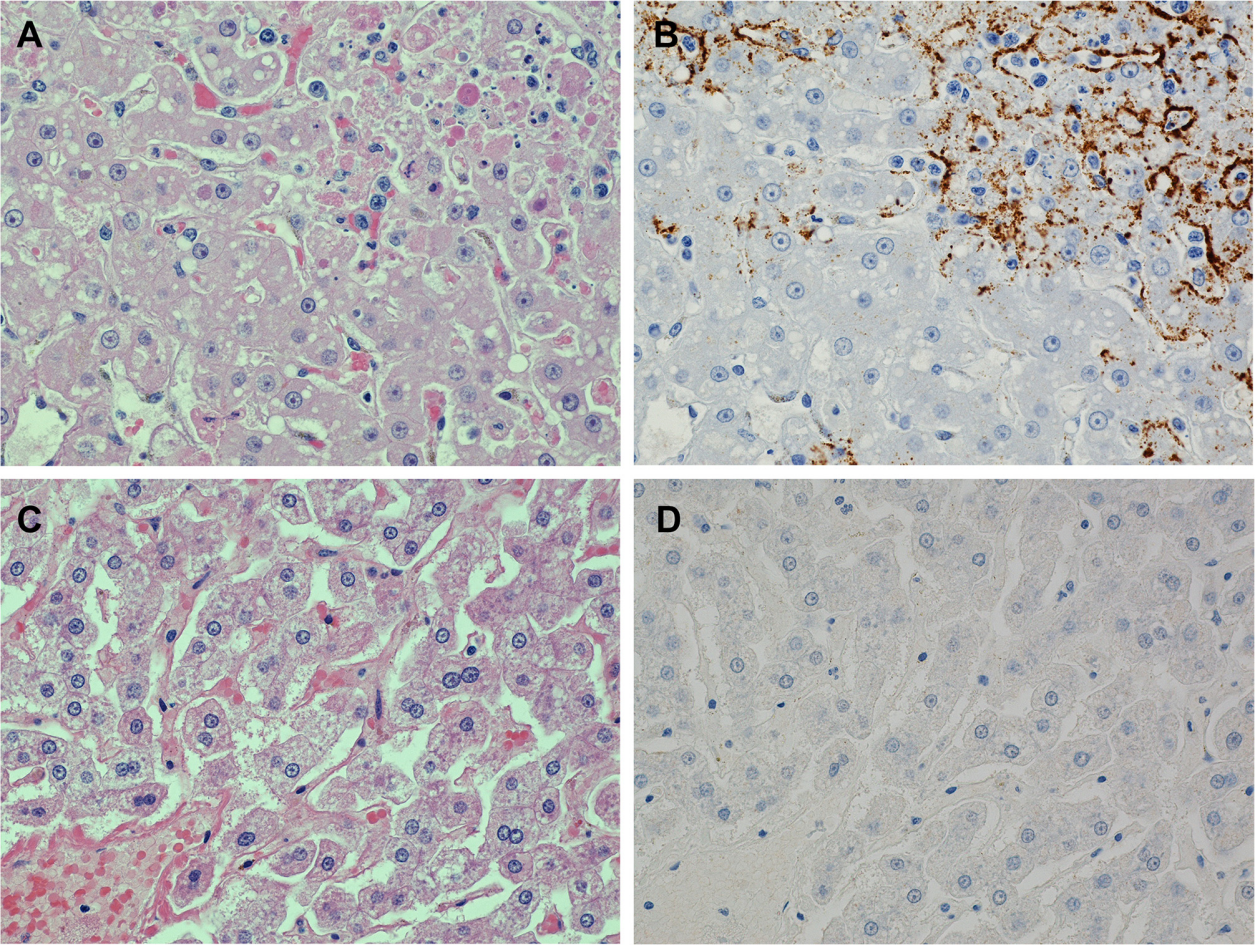
Photomicrographs of liver from two macaques that were challenged with MARV. (A) Liver from a control animal that died on day 11 PI has an area of hepatocellular degeneration and necrosis (upper right corner). (B) IHC of a replicate section of area shown in A demonstrating abundant brown-staining MARV antigen in the lesion. (C) Normal liver from an animal that was treated with AVI-7288 beginning 96 h PI and which survived the viral challenge. (D) IHC of a replicate section of area shown in C demonstrating that no MARV antigen is present. 40X objective magnification for all photos. Hematoxylin & eosin stain for A and C. Immunoperoxidase with hematoxylin counter-stain for B and D.

**Fig 8:**
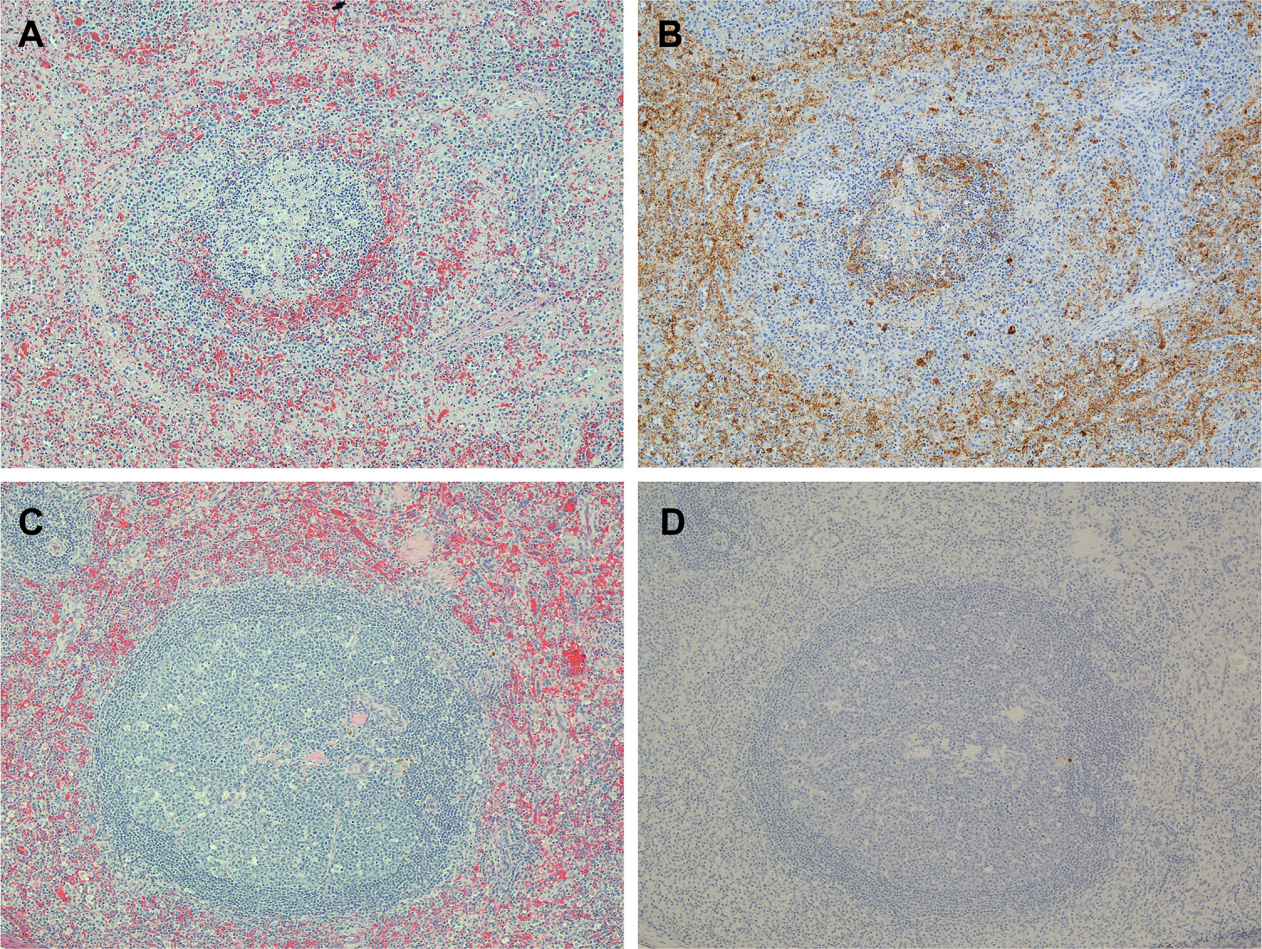
Photomicrographs of spleen from two macaques that were challenged with MARV. (A) Spleen from a control animal that died on day 9 PI has an oval lymphoid nodule (center of photo) with markedly reduced numbers of lymphocytes (i.e. lymphoid depletion); there are pale pink deposits of fibrin in the surrounding red pulp. (B) IHC of a replicate section of area shown in A demonstrating brown-staining MARV antigen in the lymphoid nodule and especially in the red pulp. (C) Lymphocyte proliferation (i.e. lymphoid hyperplasia) is present in a splenic lymphoid nodule from an animal that was treated with AVI-7288 beginning 48 h PI and which survived the viral challenge. (D) IHC of a replicate section of area shown in C demonstrating that no MARV antigen is present. 10X objective magnification for all photos. Hematoxylin & eosin stain for A and C. Immunoperoxidase with hematoxylin counter-stain for B and D.

Results from post-mortem anatomic pathology analyses indicated that all of the PBS-treated control animals developed fatal MARV infections with gross and histologic lesions and immunohistochemistry findings typical of those previously seen in cynomolgus macaques that had been experimentally infected with MARV. The organs that were consistently and most severely affected were the liver and spleen. Liver lesions consisted of multiple foci of hepatocellular degeneration and necrosis accompanied by varying degrees of acute inflammation (Fig 7A); IHC demonstrated that abundant MARV antigen was present within these lesions (Fig 7B). In the spleens, there was moderate to marked lymphoid depletion of the white pulp, usually accompanied by lysis of lymphocytes and deposition of fibrin in the adjacent red pulp (Fig 8A). Large amounts of viral antigen were present within histiocytic cells in the red and white pulp as well as in the extracellular fibrin (Fig 8B).

Only three of the 24 AVI-7288-treated monkeys failed to survive to the end of the study. One animal in the 1h PI treatment group died while under anesthesia on day 7 of the experiment and the MARV-induced lesions in its organs were very mild compared to the controls; the cause of death was attributed to anesthetic complications rather than MARV infection.

The other two AVI-7288 treated monkeys that failed to survive (one from the 24 h PI group and one from the 96 h PI group) had lesions indicating that MARV infection was the cause of their death. However, in each case there were microscopic lesions and/or IHC results that were not typical of those present in the control animals, which suggests that the AVI-7288 treatments had affected the viral infection.

In contrast to the control animals, the livers of the 21 AVI-7288 treated monkeys that survived to the end of the study had only minimal virus-induced damage and no MARV antigen was detected by IHC (Fig 7C and 7D). Rather than lymphoid depletion as occurred in the controls, there was hyperplasia of the lymphoid tissue in the spleen and other lymphoid organs of the survivors (Fig 8C); this was likely a response to systemic antigen stimulation from the viral challenge. IHC did not reveal MARV antigen in the spleen of any of the survivors (Fig 8D).

Seventeen of the 21 survivors were males and eight of these animals (from the 24, 48, and 96 h PI treatment groups) had foci of mild to moderate testicular inflammation with viral antigen in these lesions. One of these monkeys (96h PI treatment group) with immunopositive testicular lesions also had some viral antigen within acellular debris in the crypt of a palatine tonsil. One female survivor monkey (from the 1 h PI treatment group) had faint staining for viral antigen in some macrophages within foci of inflammation in the thyroid gland. No MARV antigen was detected in any tissue from any of the other 12 monkeys that survived to the end of the study.

## Discussion

MVD in humans is usually characterized by high fever, anorexia, diarrhea, petechial rash, and various hemorrhagic manifestations; these clinical signs are also reproduced in experimentally-infected cynomolgus macaques [28]. A recent report demonstrated post-exposure treatment protection of rhesus monkeys from MARV-Angola strain with treatment delay up to 3 days [7]. AVI-7288 has previously been shown to be highly effective in providing survival benefit in cynomolgus macaques infected with the Musoke strain of MARV, however, the PMO*plus* therapy in these experimental studies began on the day of infection [23]. In contrast, a treatment regimen for infected humans would usually need to be initiated in a longer post infection time period. Hence, it is important to determine the potential benefit of AVI-7288 when initiation of treatment is significantly delayed after infection. Thus, the primary objective of this study was to determine the survival benefit provided by AVI-7288 when initially administered up to four days after infection with the Musoke strain of MARV, utilizing the cynomolgus macaque animal model.

All of the control animals in this study succumbed to MARV infection; whereas, 21 of the 24 animals (87.5%) treated with AVI-7288 survived. The difference in survival rate between the control groups and the treated animals was highly significant. Furthermore, differences in survival between groups receiving AVI-7288 treatments at different times PI were not statistically distinguishable. Two of the three AVI-7288 treated animals (0197 in the 24 h group and 9172 in the 96 h group) that succumbed to infection were slow to mount an IgG immune response (1:30 and 1:270 titers, respectively). However, a low IgG titer was not adequate to provide a reliable marker of survival as several of the survivors had low IgG titers on day 14 PI. There was no notable relationship between the magnitude or duration of the MARV-specific IgG response and the time of AVI-7288 dosing or macaque survival. Overall, the results of this study indicate that, when administered IV once per day for a total of 14 days, AVI-7288 protects cynomolgus macaques against MARV even when the treatments are initiated as late as 96 h after viral infection.

Of the 21 treated macaques that survived the viral challenge, IHC revealed the presence of MARV antigen in organs of nine animals. One monkey had faint staining for viral antigen in macrophages within foci of inflammation in the thyroid gland. Although it is possible that this is an indication of persistent viral infection in the thyroid, it is more likely that these macrophages contain viral antigen due to phagocytosis of cellular debris containing viral antigen. Similarly, another animal had viral antigen in acellular debris within a tonsillar crypt. This probably reflects persistence of isolated viral antigen rather than the presence of intact, viable viral particles. However, eight of the male monkeys had testicular lesions with positive viral antigen in the foci of testicular inflammation; this might indicate that these macaques had persistent MARV infections at the time of their deaths. This is consistent with findings in humans who died of MARV during the original 1967 outbreak in which autopsy revealed large necrotic lesions in the testicles [29]. Furthermore, a man who survived MARV infection during this outbreak is believed to have infected his wife via sexual contact more than 6 weeks after his clinical recovery and viable MARV was subsequently recovered from his seminal samples as late as 16 weeks after his discharge from the hospital [30]. The possibility of venereal transmission may not be confined to MARV; a household contact of an Ebola patient from the 1995 Kikwit outbreak was presumably infected by sexual intercourse and four out of five convalescent patients examined had seminal fluid samples that were PCR positive for Ebola virus [29]. Given the on-going large Ebola virus epidemic in West Africa, sexual transmission of the virus is a concern [31]. Interestingly, although eight of the survivor male macaques in this study had MARV antigen in their testicular lesions, none of these tissues yielded viable virus by plaque assay suggesting that the treatment suppressed viral replication in the animals’ testes. Additional research needs to be done to clarify the potential for viral persistence in the testis.

In this study, we showed that the antisense compound AVI-7288 was highly effective against MARV infection. We demonstrated the efficacy of this compound administered up to four days after infection with MARV in a cynomolgus macaque animal model. It should be noted that infected animals first became PCR positive on day 5 and first showed clinical signs of disease on day 8 post infection. Administration of AVI-7288 resulted in marked improvement in survival and amelioration of laboratory and pathological manifestations of MARV disease. The current Ebola virus outbreak in West Africa reminds us of the need to identify effective antiviral products in advance of such emergency situations. As such, this is an area of filovirus therapeutics development that deserves further study, particularly to further define the limits of when treatment can be successfully initiated. Furthermore, AVI-7288 has recently been shown to be safe and well-tolerated in humans [32] and should be further developed as an effective MARV therapeutic.

## Supporting Information

S1 Fig Temperature and body weight changes. **A)** Average daily rectal temperature (°F) of macaques in the saline treatment group (filled circles; red line) and the 1 h post-infection treatment group (open circles; blue line). Fever was first observed on day 6 post-challenge. **B)** Changes in average daily body weight of macaques in the saline group (filled circles; red line) and the 1 h post-challenge treatment group (open circles; blue line).
(TIF)

S2 Fig Responsiveness scores. Changes in responsiveness scores obtained from individual animal observations (the average of two observations per day) for the saline treatment group (filled black circles) and macaques that succumbed (open circles). The scoring parameters were: 0: active; 1: mild behavioral depression; 2: moderate behavioral depression; 3: head down, hunched; 4: severe behavioral depression; 5: moribund and nonresponsive. The earliest observation of a change in responsiveness was seen on day 8 post challenge.
(TIF)

S3 Fig Serum viremia (genome copies/mL) over time. Mean serum virus genome equivalents per mL serum was determined by quantitative RT-PCR. The dotted line represents the lower limit of quantitation (LLOQ) of 1.33 x 10^5^ genome equivalents/mL.
(TIF)

S4 Fig Serum viremia (genome copies/mL) for individual macaques for days 4 through 8 post challenge. Each animal is represented by filled circles for AVI-7288 treatment groups and open circles for the saline treatment group. The dotted line represents the lower limit of quantitation (LLOQ) of 1.33 x 10^5^ genome equivalents/mL. No animals had quantifiable viral genome copies on day 4 post challenge, but all animals had quantifiable viral genome copies on day 8 post challenge. Most, but not all animals, first presented with quantifiable viral genome copies on day 5 post challenge.
(TIF)

S5 Fig Viral load in tissues. Tissue viral load of macaques exposed to MARV and treated with saline or AVI-7288 at 1 h, 24 h, 48 h, or 96 h post challenge.
(TIF)

S6 Fig Alanine aminotransferase (ALT). Analysis of serum ALT levels for macaques on day 8 post challenge.
(TIF)

S7 Fig Serum bilirubin. Analysis of serum bilirubin levels for macaques on day 8 post challenge.
(TIF)

S8 Fig Alkaline phosphatase (ALP) and gamma glutamyl transferase (GGT). Analysis of serum (A) ALP and (B) GGT levels in macaques treated with AVI-7288 96 h post challenge. Since all the animals in the saline treatment group succumbed to infection after day 10, animals in the 1 h treatment group were included for comparison.
(TIF)

S9 Fig Platelets and MPV. Analysis of (A) platelets and (B) MPV from macaques in all treatment groups on day 8 and day 10, respectively, post challenge.
(TIF)

S10 Fig Coagulation. Analysis of serum (A) activated partial thromboplastin time (APTT) and (B) prothrombin time (PT) over the course of the study. Since all the animals in the saline treatment group succumbed to infection after day 10, results from the 1 h and 96 h treatment groups were compared.
(TIF)

S11 Fig Cytokines. Analysis of the cytokines (A) IL-6, (B) MDC, (C) MIP-1β, and (D) TARC from macaques in all treatment groups on day 8 post challenge.
(TIF)
