# Supplementary figures and images for: Delayed Time-to-Treatment of an Antisense Morpholino Oligomer Is Effective against Lethal Marburg Virus Infection in Cynomolgus Macaques
Source: PLoS Negl Trop Dis. 2016 Feb 22;10(2):e0004456. doi: 10.1371/journal.pntd.0004456 (PMC4764691; doi:10.1371/journal.pntd.0004456)

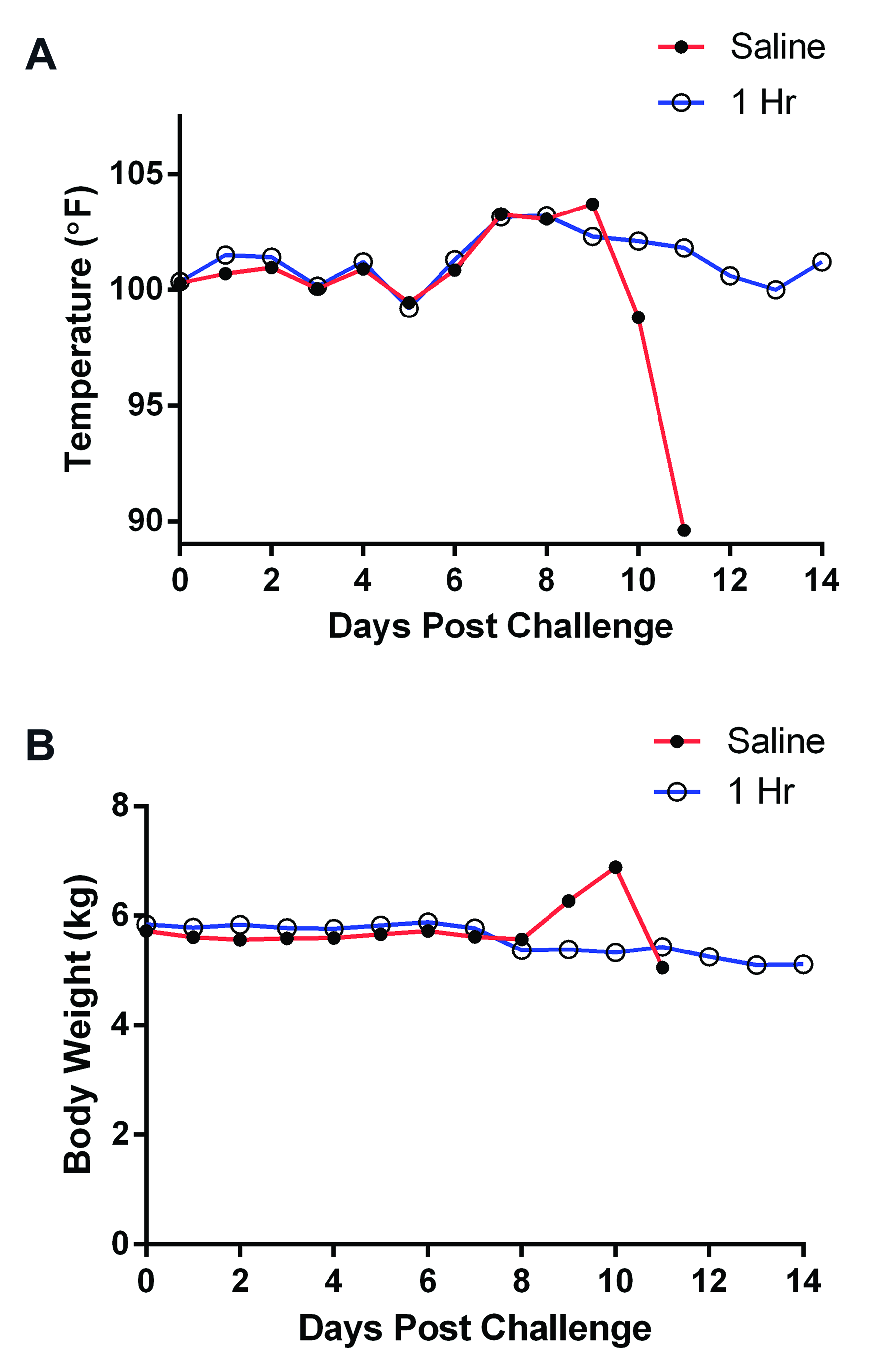

Supplement: S1 Fig — A) Average daily rectal temperature (°F) of macaques in the saline treatment group (filled circles; red line) and the 1 h post-infection treatment group (open circles; blue line). Fever was first observed on day 6 post-challenge. B) Changes in average daily body weight of macaques in the saline group (filled circles; red line) and the 1 h post-challenge treatment group (open circles; blue line). (TIF) [file pntd.0004456.s001.tif]
